# Recommendations for acquisition, interpretation and reporting of whole body low dose CT in patients with multiple myeloma and other plasma cell disorders: a report of the IMWG Bone Working Group

**DOI:** 10.1038/s41408-018-0124-1

**Published:** 2018-10-04

**Authors:** Lia A. Moulopoulos, Vassilis Koutoulidis, Jens Hillengass, Elena Zamagni, Jesus D. Aquerreta, Charles L. Roche, Suzanne Lentzsch, Philippe Moreau, Michele Cavo, Jesus San Miguel, Meletios A. Dimopoulos, S. Vincent Rajkumar, Brian G. M. Durie, Evangelos Terpos, Stefan Delorme

**Affiliations:** 1 First Department of Radiology, National and Kapodistrian University of Athens, School of Medicine, Athens, Greece; 2 Department of Medicine, Roswell Park Comprehensive Cancer Center, Buffalo, NY USA; 3 Seràgnoli Institute of Hematology, Bologna University School of Medicine, Bologna, Italy; 4 Department of Radiology, Clinica Universidad de Navarra, Pamplona, Spain; 5 Department of Radiology, Roswell Park Comprehensive Cancer Center, Buffalo, NY USA; 6 Division of Hematology/Oncology, Columbia University Medical Center, New York, NY USA; 7 Department of Hematology, University of Nantes Health Centre, Nantes, France; 8 Clinica Universidad de Navarra-Centro de Investigación Médica Aplicada, Instituto de Investigación Sanitaria de Navarra, Centro de Investigación Biomédica en Red de Cáncer, Pamplona, Spain; 9 Department of Clinical Therapeutics, National and Kapodistrian University of Athens, School of Medicine, Athens, Greece; 10 Division of Hematology, Department of Internal Medicine, Mayo Clinic, Rochester, MN USA; 11 Samuel Oschin Comprehensive Cancer Institute, Cedars Sinai Outpatient Cancer Center, Los Angeles, CA USA; 12 Department of Radiology, German Cancer Research Center, Heidelberg, Germany

## Abstract

Whole Body Low Dose CT (WBLDCT) has important advantages as a first-line imaging modality for bone disease assessment in patients with plasma cell disorders and has been included in the 2014 International Myeloma Working Group (IMWG) criteria for multiple myeloma (MM) definition. Nevertheless, standardization guidelines for the optimal use of WBLDCT in MM patients are still lacking, preventing its more widespread use, both in daily practice and clinical trials. The aim of this report by the Bone Group of the IMWG is to provide practical recommendations for the acquisition, interpretation and reporting of WBLDCT in patients with multiple myeloma and other plasma cell disorders.

## Introduction

Bone disease is one of the hallmarks of multiple myeloma (MM), and it occurs as a result of increased osteoclast activity and suppressed osteoblast function, leading to enhanced bone resorption^1^. Up to 80% of newly diagnosed MM patients present with osteolytic lesions, increasing the risk for skeletal-related events, which in turn are associated with increased morbidity and mortality^2^. The definition of bone disease in MM has evolved over the years reflecting, among others, significant advances in imaging technology. For decades, myeloma-related bone disease was defined as the presence of osteolytic lesions or the presence of osteoporosis due to myeloma^3^. Traditionally, the conventional skeletal survey (CSS), consisting of a series of plain radiographs of the axial skeleton and proximal limbs, was used to assess the presence of the above findings. The major drawback of conventional radiographs is their significantly lower sensitivity compared to cross-sectional imaging techniques for the diagnosis of osteolytic lesions^4^. For example, it has been shown that detection of osteolyses on lateral x-rays of the lumbar spine is only possible when between 50 and 75% of the cancellous bone thickness has been replaced^5^. Although conventional radiographs were still the preferred modality for the definition of bone disease in the 2003 International Myeloma Working Group (IMWG) criteria for the diagnosis of MM, the authors added that CT or MRI could be used to clarify findings^6^. In the 2014 updated IMWG criteria for the diagnosis of MM, it was stated for the first time that the presence of one or more osteolytic bone lesions seen on CT or PET-CT does fulfill the criteria for bone disease, regardless of whether they are seen on conventional radiographs or not^7^. Furthermore, in view of the incorporation of these more sensitive cross-sectional imaging modalities, the presence of osteoporosis alone, in the absence of lytic lesions or in the absence of focal lesions on MRI, is no longer sufficient evidence of bone disease per the updated diagnostic criteria for MM.

Whole body MRI is gaining momentum for the assessment of patients with plasma cell neoplasms, due to whole body coverage, excellent sensitivity for bone marrow involvement without bone destruction, and incorporation of advanced techniques such as diffusion-weighted imaging and Dixon-based fat fraction evaluation^8–10^. Nevertheless, many issues regarding availability, cost, standardization of technique and radiologic expertise need to be addressed for it to become more widely accepted as the imaging modality of choice in all stages of patient management in MM, including initial assessment. On the other hand, there are definite advantages in the use of whole body low dose CT (WBLDCT) as a first-line imaging modality in newly diagnosed patients with MM. It is widely available, relatively cheap, simple to perform and patient-friendly with a scanning time of less than a minute.

The inclusion of osteolytic lesions found on CT alone, in the so-called myeloma-defining events according to the novel IMWG criteria for MM, was the result of multiple studies showing that CT has a greater sensitivity than the CSS for detection of multiple myeloma bone lesions. Most of these studies have focused on WBLDCT protocols, which offer the advantage of both whole-body coverage and lower effective radiation dose delivered to the patient^11–15^. Although low dosage comes at the cost of reduced image quality overall (mainly owing to image noise), the study of the skeleton is not significantly impaired due to the intrinsic contrast between high-density mineralized bone and soft-tissue density osteolytic lesions^4^.

The IMWG recently published the results from the largest study directly comparing WBCT and CSS, in 212 patients with newly diagnosed MM from eight international centers^16^. It showed, not only that WBCT is significantly more sensitive than CSS for detecting myeloma-related osteolyses, but also that lytic lesions detected by WBCT alone are prognostically significant for the progression of smoldering MM into MM. However, the imaging protocols implemented in the various centers involved in the study and retrospectively analyzed by some of the authors of the present article (L.A.M., V.K., S.D.), differed in many regards (e.g., tube current, placement of the arms, slice thickness, and others), highlighting the fact that there is currently no published consensus on the optimal technical requirements for WBCT in MM. Although most of the WBCT exams reviewed in that study were acquired using a low dose protocol, some were not. Moreover, in a number of cases the WBCT exam was performed as part of a PET-CT protocol. The absence of guidelines for the optimal use of WBLDCT in myeloma may be one of the reasons for the persistence of the CSS, especially outside major academic centers, despite the much lower sensitivity of plain radiographs for assessing bone disease. The aim of this article is, therefore, to provide practical recommendations for the acquisition, interpretation and reporting of WBLDCT studies in patients with multiple myeloma and other plasma cell neoplasms.

## Acquisition of images

As early as 1985, Schreiman et al. showed that standard-dose CT reveals more lesions than the conventional skeletal survey in patients with MM^17^. However, applying a standard-dose whole-body CT protocol results in very high cumulative radiation doses. Mahnken et al used a conventional high-dose CT of the thoracic and lumbar spine to assess bone disease in MM patients, and calculated that the effective dose ranged between 25.5 and 36.6 mSv^18^. In 2005, Horger et al were the first to establish the feasibility of a whole body low dose multidetector CT protocol in the diagnosis of MM, as an alternative to conventional X-rays^19^. They used standard tube voltage (120 kV) with very low tube current (time-current products of 40, 50, 60, and 70 mAs), and rated image quality using a four-point scale (very good, good, sufficient for diagnosis, insufficient). The time-current product did not affect the image quality significantly and only the “very good” ratings changed to “good” as less energy was applied. The effective radiation dose ranged from 4.1 mSv to 7.5 mSv depending on the tube current. In another study, Kropil et al used a tube voltage of 100 kV and an effective time-current product of 100 mAs, with automatic tube current modulation enabled. Both for female and male patients an effective dose of approximately 4.8 mSv was calculated using these parameters^12^. The most exhaustive comparison of the effect of dose parameters on bone image quality at WBLDCT was performed by Gleeson et al., who applied 14 different WBLDCT protocols on a single cadaver, using various combinations of tube voltage (ranging from 80 to 140 kV) and time-current product (ranging from 14 to 125 mAs)^20^. Image quality of each data set was rated on a five-point scale ranging from “very good” to “poor and/or nondiagnostic”. Among the combination of tested parameters (kV/mAs), the higher-rated for image quality were 100/80, 140/35 and 120/50, at an average effective dose of around 4.5 mSv. The authors however, selected 140 kV/14mAs with automated tube current modulation activated, and a moderately sharp reconstruction algorithm, as the optimum parameters because they produced diagnostic images with a very low effective dose (1.74 mSv), comparable to that of a conventional skeletal survey (most estimations of effective radiation dose for a conventional skeletal survey vary between 1.5 and 2.5 mSv, depending on patient weight and equipment)^4,12,14^. From the above data, it is clear that different approaches can be used in order to produce bone images of acceptable diagnostic quality, while keeping radiation doses at low levels.

In this section, we present recommendations on the technical parameters of a WBLDCT scan, as well as on proper patient positioning.

### Technical parameters

The WBLDCT exam should be performed on a multidetector scanner with 16 detector rows or more, allowing for very short scanning times and acquisition of thin slices, without radiation dose penalty. Scan coverage must be from the cranial vault to at least the proximal metaphysis of the tibia. As stated above, different combinations of tube voltage and current have been used by various investigators, and no validated data exist relative to the optimal parameters^4^. Based on the evidence available, we suggest the use of a 120 kV voltage and a time-current product between 50 and 70 mAs, but we do recognize that other approaches (e.g., 140 kV with 14–25 mAs) may be equally effective. A narrow collimation protocol with optimal reconstruction algorithms should be used. The collimation must be set between 0.5 and 1.5 mm, depending on scanner type. Iterative reconstruction algorithms, which are currently offered with most modern scanners, allow for a substantial reduction of both image noise and artifacts and should be always used, if available^21^. Some investigators reconstruct the thinner collimation data using only one intermediate, middle-frequency kernel, appropriate for viewing all images using different window settings^11,19^. Another approach is to reconstruct images using both a sharp, high spatial frequency convolution kernel (“bone” algorithm), and a smoother convolution kernel for soft tissue assessment. The sharpest images have increased spatial resolution and are optimal for detection of small osteolyses. Since sharp reconstruction algorithms can also increase the background noise in the image, they should preferably be used in protocols with a tube time-current product of more than 50 mAs^19^. The soft tissue reconstruction algorithm images are used for assessment of paramedullary and extramedullary soft tissue masses. They are also optimal for the evaluation of focal and diffuse hyperdense myeloma deposits in the medullary cavities of the long bones. The soft tissue convolution kernel is also needed if Hounsfield unit (HU) measurements of medullary hyperdensities are to be performed. Some authors have used such measurements to distinguish peripheral myeloma deposits from normal or hyperplastic red marrow rests as well as for prognostic purposes^22,23^. However, it is well known that CT attenuation measurements are influenced by multiple parameters including the convolution kernel used^24,25^. Therefore, CT measurements of peripheral intramedullary lesions should always be performed on the soft tissue reconstructed images, and not on the soft tissue window settings of the bone algorithm images. Ideally, HU thresholds, specific for a scanner-convolution kernel combination, should be investigated and scanners should be calibrated regularly using phantoms to maintain uniformity of measurements over time. The axial slice thickness of the reconstructions varies in the literature between 1 and 5 mm. We consider an axial slice thickness reconstruction of 2 or 3 mm reasonable, provided that the slice increment (i.e., the distance between the centers of adjacent slices) is set at a lower value than the slice thickness, which results in overlap between consecutive slices. For example, axial slices can be reconstructed with 2 mm thickness and 1 mm increment, or 3 mm thickness and 1.5 mm increment, resulting in both cases in 50% overlap between adjacent slices. This approach produces axial slices that are generally adequate even for the detection of small osteolytic lesions, and can also serve as source images for the extraction of good quality sagittal and coronal multiplanar reformations (MPRs)^19,22,26^. The MPRs can be easily generated using either standard software provided by most CT scanner vendors, or third-party diagnostic software. Sagittal MPRs of the bone algorithm images provide an excellent overview of the whole spine, permitting detection of vertebral compression fractures (VCFs), which can be easily missed on the axial slices. They may also help characterization of VCFs (benign versus malignant), and estimation of spinal fracture risk. Additional MPRs reconstructed parallel to the long axis of the femora and humeri are helpful for evaluating the medullary cavity for the presence of focal or diffuse hyperdense myeloma deposits. They facilitate measurement of the size and extent of such deposits and the detection of severe endosteoal scalloping which may increase fracture risk. The proposed technical parameters are summarized in Table 1.

**Table 1:** Technical parameters for WBLDCT exams in MM

Number of detector rows	16 or more
Scan coverage	Cranial vault to proximal tibial metaphysis (include humeri in the field of view)
Tube voltage(kV)/time-current product (mAs)	120/50–70^a^
Collimation	0.5–1.5 mm
Reconstruction convolution kernel	Sharp, high-frequency (bone) and smooth (soft tissue). Alternatively, one middle-frequency kernel for all images
Iterative reconstruction algorithms	Yes (to reduce image noise and streak artifacts)
Thickness/increment of axial slices	2/1 mm or 3/1.5 mm
Multiplanar Reconstructions (MPRs)	Yes (sagittal, coronal and parallel to long axis of proximal limbs).

^a^Different tube parameters (e.g., 140/14–25 or a low voltage approach) are acceptable as long as they produce images of diagnostic quality with low effective patient dose

### Patient preparation and positioning

No patient preparation is necessary before the exam and there is no need for oral or intravenous contrast administration. The patient should lie supine on the table with the arms placed in such a position as to include the humeri in the field of view, without producing artifacts that could degrade significantly the image quality in clinically relevant (i.e., skeletal) anatomic regions. This can be achieved with one of two positionings: In the first, the patient’s arms are placed above the head with as little bending at the elbows as possible. This way, the entire humeri can be included in the field of view and the reconstruction of MPR views along the axis of the bone shaft (which is particularly useful for the evaluation of the medullary cavities) is greatly facilitated. This positioning of the upper extremities can produce a few beam hardening artifacts superimposed on the skull, but these are almost never significant and they do not pose a diagnostic problem. Care should be taken not to bend the elbows too much, since this can result in a significant portion of the humeri lying ouside of the field of view or at the edges of it, where truncation artifacts may degrade image quality significantly. Alternatively, the upper extremities can be positioned alongside the body. In this case, the arms should not lie on the table, because this places them at the level of the spine and streak artifacts from beam hardening, which are inevitably produced with low dose protocols, may cause marked reduction in the image quality of the thoracic and lumbar vertebrae, especially in overweight or obese patients. Instead, the upper extremities should lie in front of the body with the hands folded. In this way, the arms are placed in an elevated position, and not at the same level as the spine, therefore any artifacts as a result of beam hardening will be produced more anteriorly and, thus, will not be superimposed on the thoracic and lumbar spine. It must be noted that, whereas one of the two described arm positionings can be maintained by most patients during the very short study time of a WBLDCT scan, this may not be so during the longer duration of a PET/CT scan. Therefore, the CT part of a PET/CT study may suffer from limitations due to the placement of the upper extremities alongside the body. In such cases a custom-made arm rest may be helpful.

**Fig. 1:**
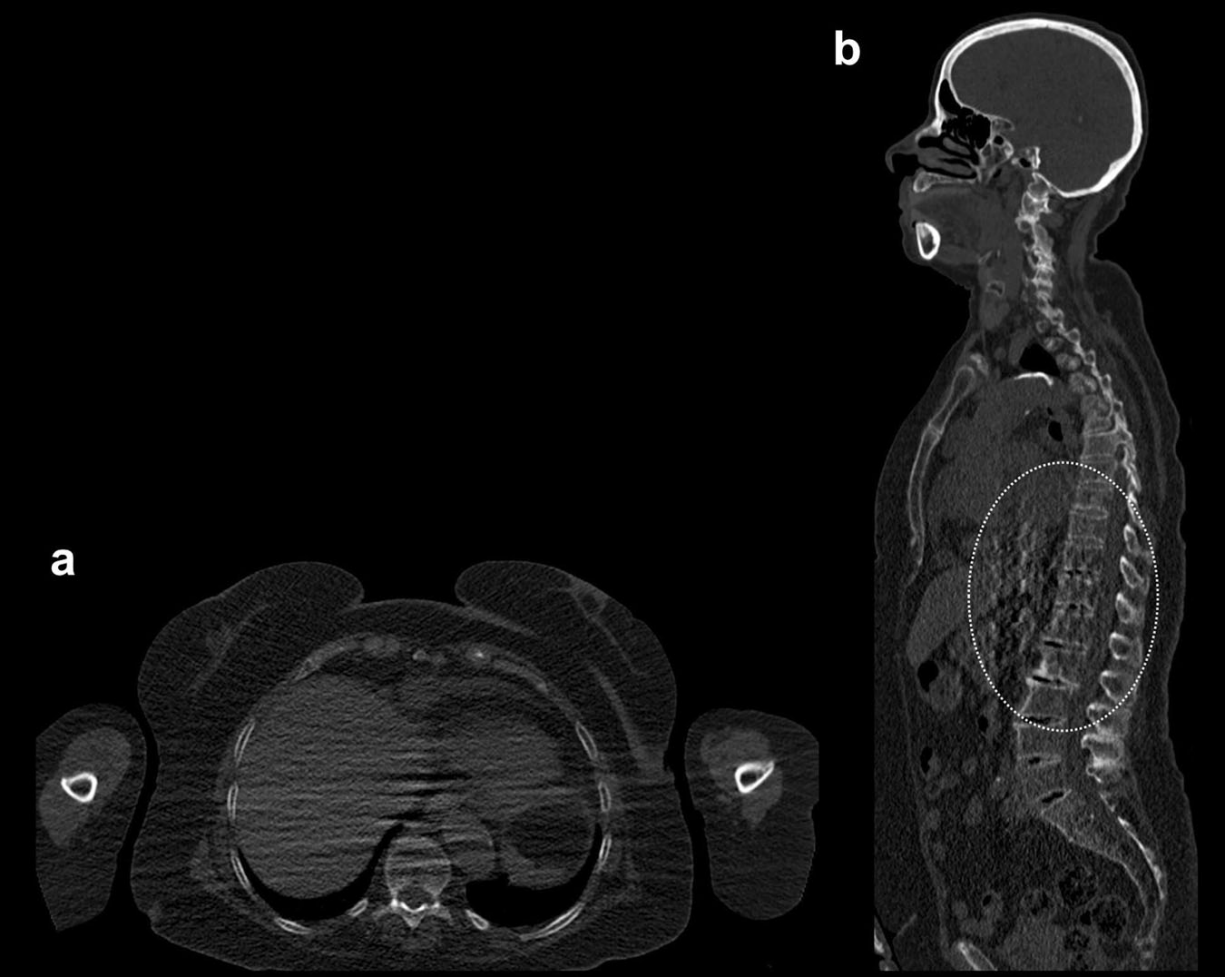
WBLDCT scan (120 kV, 60 mAs) of a 76-year-old female with newly diagnosed smoldering myeloma, performed on a 16-slice CT scanner. **a** Axial slice (2/1 mm thickness/increment) at the level of the T10 vertebral body. The arms are positioned alongside the body. Assessment of the vertebra is significantly impaired due to the presence of multiple superimposed streak artifacts from beam hardening. **b** The artifacts are also very prominent on the sagittal MPR image, mainly at the level of the lower thoracic spine (dotted ellipse). *MPR* multiplanar reformation

**Fig. 2:**
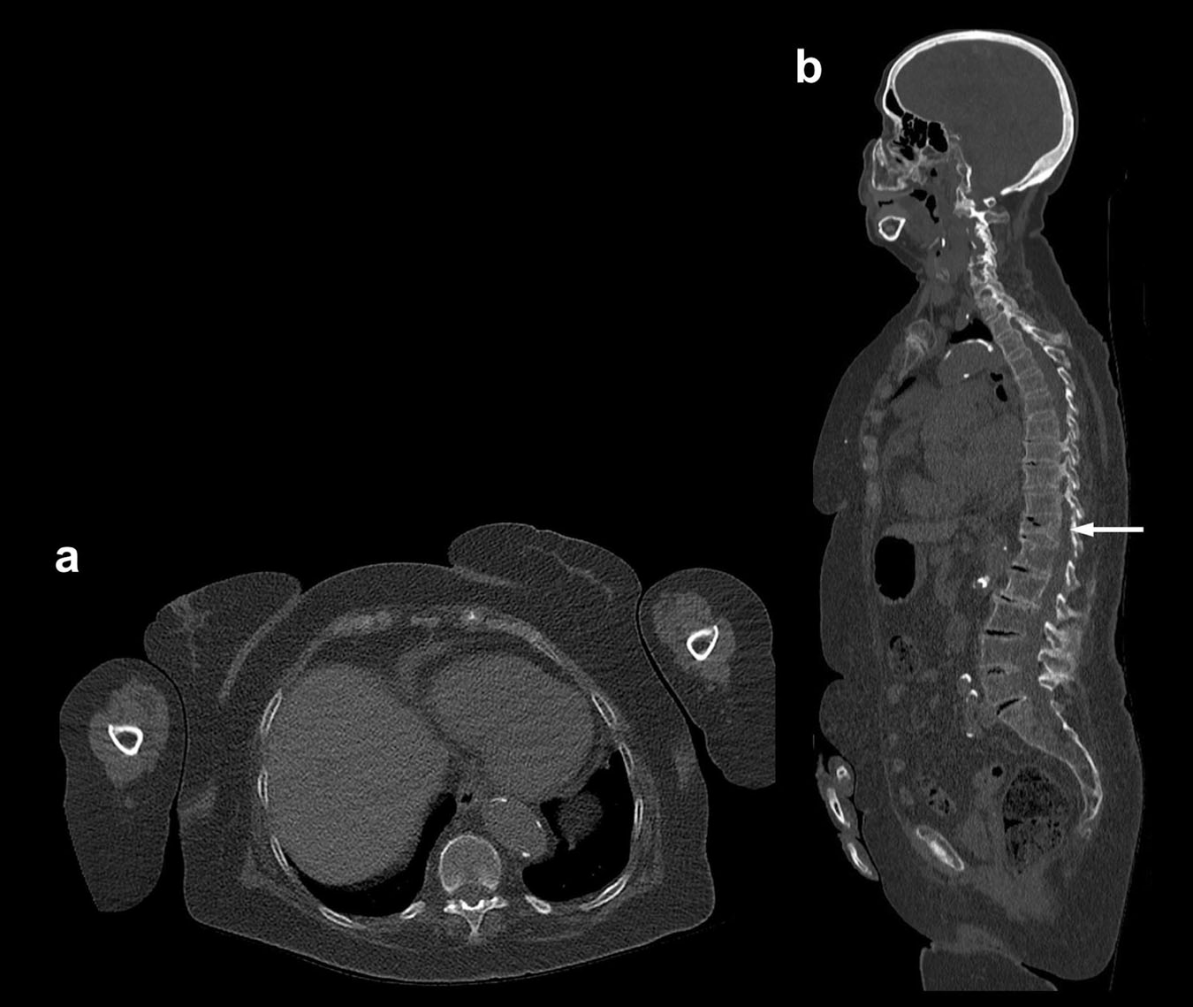
WBLDCT scan of the same patient as in Fig. 1, performed on a 128-slice CT scanner three years later with the same tube voltage and time-current product (120 kV, 60 mAs). The arms are lying more anteriorly than in Fig. 1, and an iterative reconstruction algorithm was used to reduce artifacts and image noise. **a** Axial slice (2/1 mm thickness/increment) at the level of the T10 vertebral body and **b** Sagittal MPR image. Overall image quality is significantly improved compared to the images of Fig. 1. The trabecular structure of the T10 vertebral body is readily appreciated in **a** and spinal anatomy is well depicted on **b**. Note new compressive vertebral fracture of T11 (arrow in **b**). *MPR* multiplanar reformation

An example of the effect of scanner hardware, reconstruction algorithm type and patient positioning on image quality is shown in Figs. 1 and 2.

## Interpretation of imaging findings

Thin-slice coverage of the whole skeleton produces a very large set of images, which must be reviewed in an orderly pattern, so as to decrease the likelihood of missing or misinterpreting findings. Although we recognize that alternative approaches may be equally valid, one suggestion for structured image viewing is as follows: the bone algorithm axial images should be reviewed first, in descending order, beginning from the skull vertex and then proceeding to the cervical, thoracic and lumbar spine, the pelvic bones, and the lower limbs. Then, scrolling images in the opposite direction, the ribs, sternum, scapulae, clavicles and upper limbs are assessed. Sagittal MPRs of the spine should be reviewed next, to diagnose and characterize vertebral compression fractures and to evaluate vertebral fracture risk. Following that, the presence or absence of focal and/or diffuse intramedullary hyperdensities of the femora and humeri is examined using both axial images and MPRs. Finally, visceral assessment using soft tissue images should be performed, to look for extramedullary disease or non-osseous incidental findings^27^. The above described viewing order proved to be suitable for complete whole-body assessment in a reasonable time frame during the analysis of the cases of the comparative WBCT/CSS IMWG study^16^.

### Osteolytic lesions

Myeloma-related osteolyses appear as focal destructive lesions of the cancellous (trabecular) bone, without a sclerotic border (unless previously treated). They are easily seen on low dose CT protocols against well mineralized trabecular bone, although in some patients with marked osteoporosis they may be difficult to discriminate from areas with rarefied trabeculae. Even very small lesions can be picked up by thin-collimation CT due to its superior spatial resolution, although we propose that only osteolytic lesions with a diameter of 5 mm or more should be taken into account. We acknowledge that this is an arbitrary cut-off, well above the resolution capabilities of modern CT scanners, but we consider it useful in order to avoid over-interpretation of tiny hypodense lesions of the trabecular bone. By lowering the threshold below 5 mm, there is an increased risk that very small hypodense areas that are not myeloma-related will be considered positive. Such non-specific alterations of trabecular bone are often so tiny that reliable characterization by either visual inspection or CT attenuation measurements is not possible. Nevertheless, we recommend that in the case of small suspicious lesions that do not fulfill the above strict size criteria, or larger but equivocal lesions, radiologists should exercise good judgment and recommend further imaging workup with MRI, or a repeat CT study in 3 to 6 months, depending on the clinical context.

Osteolytic lesions may extend to the cortex, causing cortical thinning or even frank destruction, which can be readily appreciated on WBLDCT. Extraosseous soft tissue masses (i.e., paramedullary disease) may accompany cortical destruction and can also be seen on WBLDCT, although very early extraosseous soft tissue extension can be imperceptible on the low dose CT images. MRI is the modality of choice in this setting, due to exquisite soft tissue contrast, and should be suggested whenever spinal cord compromise is suspected.

Not all hypodense lesions of the trabecular bone represent osteolyses. The most common false positives are fat-containing foci, probably representing benign processes such as focal yellow marrow deposits, small hemangiomas and Modic 2 type degenerative vertebral endplate changes. The presence of fat can be appreciated either visually, using subcutaneous fat as an internal standard or, in equivocal cases, by performing density measurements. It must be noted that the presence of fat excludes the diagnosis of osteolysis only in newly diagnosed non-treated MM patients, whereas in previously treated patients healed lesions can display partial or total fatty replacement. Other signs of responding lesions on WBLDCT include a decrease in size and the appearance of sclerosis (peripheral and/or central), the latter most commonly seen in patients treated with bortezomib^28^.

### Peripheral medullary lesions

Medullary infiltration without osteolysis is very hard to detect in the axial skeleton due to the presence of dense trabecular bone. On the other hand, myeloma deposits in the femora and humeri are easily depicted by WBLDCT as hyperdense lesions against the low-density background of fat containing yellow marrow which normally fills the medullary cavity of long bones in adults. Myeloma deposits in the medullary cavities of the appendicular skeleton can appear as either well-defined hyperdense nodules or as a diffuse increase in density filling part (proximal rather than distal), or the whole of the cavity. It must be stressed that not all hyperattenuating medullary regions within the appendicular skeleton represent myeloma lesions. Increased red (hematopoietic) marrow reconversion, either related to chronic causes such as severe anemia, obesity and heavy smoking or to treatment-induced changes (i.e., hematopoietic recovery phase following chemotherapy, administration of hematopoietic growth factors), may also manifest as increased density within the cavities of the proximal long bones^29^. Such areas of red marrow, usually appear more ill-defined than myeloma lesions, with generally lower mean densities. Unfortunately, no established consensus currently exists on the optimal cut-off density values to distinguish the two conditions and large cohort studies with good reference standards are warranted. The assessment of these peripheral medullary deposits on WBLDCT is essential for a number of reasons. It has been shown that they are associated with high tumor burden, advanced disease stage and poorer prognosis in patients with symptomatic myeloma^30^. In another study, they were associated with significantly elevated serum levels of paraprotein and b_2_-microglobulin at initial diagnosis^22^. Size measurements of a minimum of two peripheral medullary lesions was also found to be sufficient for response assessment and correlated very well with the course of lytic bone lesions and that of hematologic parameters^23^. Finally, there is evidence that about one third of MM patients and a diffuse MRI pattern of involvement display only abnormal hyperdensities in the medullary cavities of the appendicular skeleton and no osteolyses on WBLDCT^31^.

### Fractures

WBLDCT is excellent for the depiction of fractures, including vertebral compression fractures, as well as for the estimation of fracture risk. Vertebral fractures are best seen on the sagittal MPR images. Although MRI is the imaging modality of choice for the characterization of an acute vertebral fracture as benign (osteoporotic) or malignant, several valuable signs have been described on CT as well^32^. CT findings associated with a malignant vertebral fracture are destruction of the cancellous bone of the vertebral body with or without destruction of the cortex, destruction of a pedicle, a focal paravertebral soft tissue mass, and an epidural soft tissue mass. The presence of air within the vertebral body (intravertebral vacuum cleft sign), retropulsion of a bone fragment into the spinal canal and a thin (less than 10 mm) diffuse circumferential paraspinal soft tissue mass are CT signs indicative of acute osteoporotic fractures. Vertebral fracture risk is usually classified according to the volume of osteolysis and its location within the vertebra^33,34^. Cases where more than 50% of the vertebral body is destroyed and/or critical parts such as the costovertebral junction or the pedicle are involved, are considered as high risk and should be referred for local treatment.

**Fig. 3:**
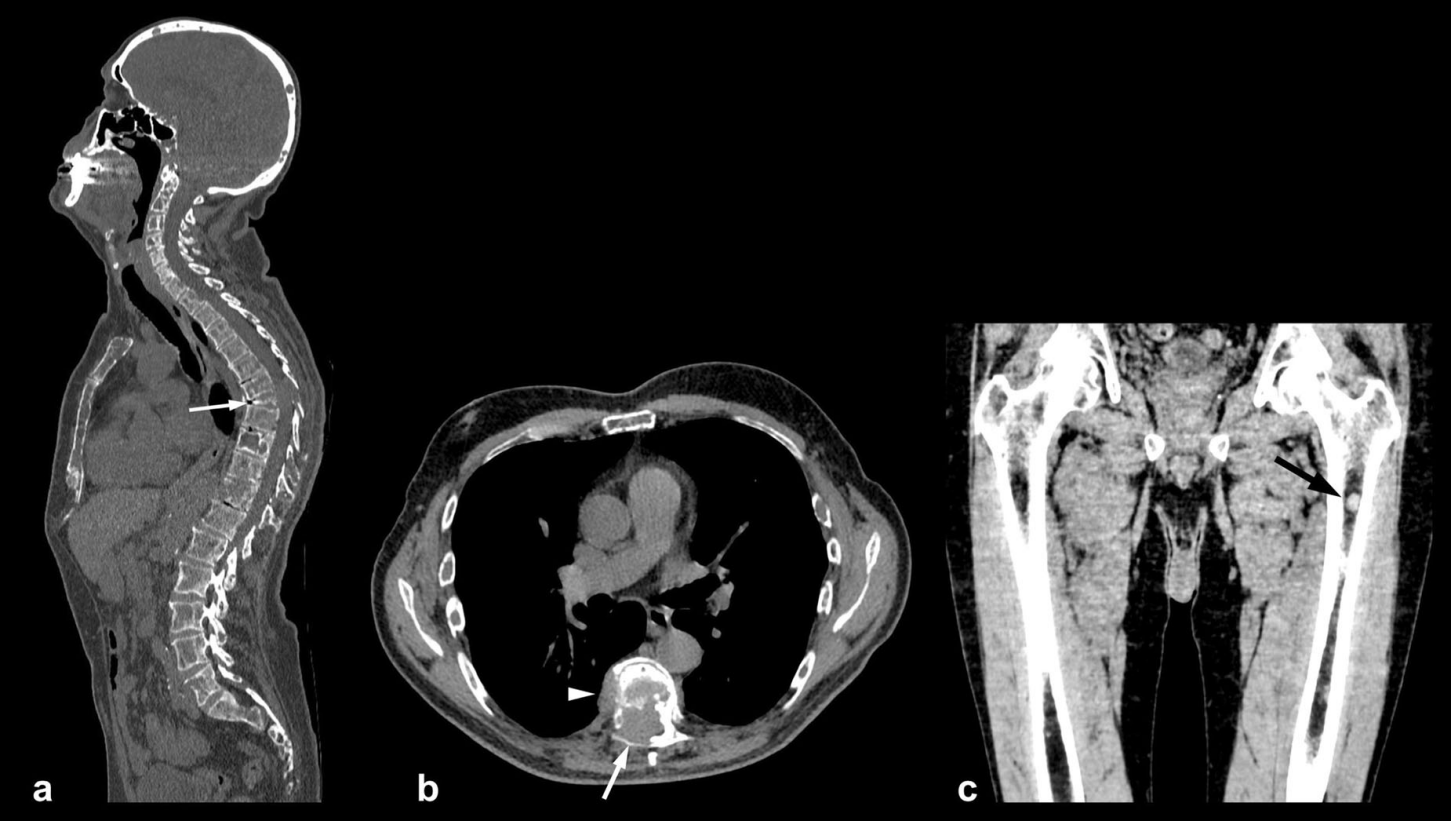
WBLDCT-based comprehensive bone disease assessment in a 65-year-old male with newly diagnosed multiple myeloma. Multiple osteolytic lesions are seen in the skull, spine and sternum on a sagittal MPR image (**a**). T7 demonstrates complete collapse (arrow in **a**). In the axial image at the level of T7 (**b**) extensive paramedullary soft tissue disease is seen inside the spinal canal (arrow) as well as in the right paravertebral space (arrowhead). In a coronal MPR image of the left femur (**c**) a 1.3 cm well-defined hyperdense nodule (mean density 80 HU) is seen in the proximal medullary cavity (arrow). *HU* Hounsfield units

An example of comprehensive bone assessment with WBLDCT in a newly diagnosed myeloma patient is shown in Fig. 3.

## Reporting of imaging findings

Structured reporting of imaging findings by the radiologist is strongly encouraged, since it facilitates communication between specialists and aids timely patient management decisions. A template for a structured report of WBLDCT findings in MM should include the following:

### Technique/Clinical information/Prior imaging studies

A few details about the technique used should be given, including number of detectors, slice thickness, anatomy scanned (e.g., skull to proximal tibial metaphyses), and whether MPRs were performed. If prominent artifacts degrade significantly the image quality in certain parts of the anatomy, this should be specifically stated. A brief summary of the patient’s clinical status should be given. Any available prior imaging studies that are relevant must be mentioned.

### Imaging findings

Findings of myeloma-related bone disease (i.e., osteolyses and peripheral medullary lesions) should be described first. Reporting of osteolyses in an orderly, structured pattern (skull to tibia in descending order) is strongly encouraged, although other approaches (e.g., largest or most clinically significant lesion first) can also be used. Size measurements of main lesions should also be performed. Increased fracture risk due to the presence of extensive osteolysis, especially in weight-bearing bones such as those of the lower spine and lower limbs should be mentioned. All extraosseous soft tissue masses must be described, and the largest of these should be measured. Special mention must be made of extraosseous disease that could cause compression of neural elements in the spinal canal and/or the intravertebral foramina. If indicated, the radiologist should suggest further work up with MRI in such cases. Intramedullary deposits in the cavity of the femora and humeri should then be described. In the case of focal intramedullary hyperdense lesions, the location, size, density and presence/absence of significant endosteal scalloping must be recorded. The same features should be noted when diffuse intramedullary hyperdensities are seen. In the latter case location/size may be described by dividing the medullary cavity in thirds: e.g., proximal third diffuse disease, or packed marrow with diffuse hyperdensities located within the whole cavity. Significant cortical scalloping/thinning caused by focal or diffuse intramedullary deposits in the long bones can increase the likelihood of fracture, especially in weight-bearing bones like the femora, and should be explicitly stated. All vertebral compression fractures must then be mentioned and morphological features described in the previous section should be used to state whether they are more likely to be benign or malignant. A comment may be added regarding the overall bone mineral density. Finally, clinically relevant incidental findings in the brain, head and neck, thorax, abdomen and pelvis should be described and clear suggestions for further work up should be given, if indicated.

### Conclusion of the report

A clear summary statement should highlight the most important findings regarding overall disease status. It should include number and distribution of osteolyses, presence/absence of extraosseous soft tissue masses, likelihood of cord/nerve root compression, number of focal medullary deposits in the appendicular skeleton, presence/absence of diffuse medullary disease in the appendicular skeleton and a comment on vertebral compression fractures and/or vertebral fracture risk. Regarding osteolyses, since there are no validated data yet on the prognostic significance of the number of lytic lesions detected by WBLDCT, an exact number should be given for up to 10 lesions only. Appropriate recommendations regarding short-term follow up or alternative imaging studies should be also made in the case of equivocal findings. Lastly, any incidental finding that raises concern should be mentioned, along with the appropriate recommendation.

## Summary

WBLDCT has important advantages as a first-line imaging modality for the assessment of bone disease in patients with plasma cell neoplasms. It has been proven to be more sensitive than the conventional skeletal survey for the detection of osteolyses, and lytic lesions detected by WBLDCT justify initiation of treatment in patients with otherwise asymptomatic disease. Selection of optimal imaging parameters and proper patient positioning are mandatory, to ensure the acquisition of images of diagnostic quality, while maintaining a low effective radiation dose delivered to the patient. A systematic approach to the interpretation of myeloma-related bone disease, including osteolyses, peripheral intramedullary lesions and fractures is advised and potential pitfalls should be acknowledged. Structured reporting facilitates reading and communication of findings, and should include a clear conclusion as well as appropriate recommendations for further workup, where indicated.
